# The evolutionary process of mammalian sex determination genes focusing on marsupial *SRY*s

**DOI:** 10.1186/s12862-018-1119-z

**Published:** 2018-01-16

**Authors:** Yukako Katsura, Hiroko X. Kondo, Janelle Ryan, Vincent Harley, Yoko Satta

**Affiliations:** 1 Department of Biology, The Pennsylvania State University, State College, USA; 2 Department of Biology, Temple University, Philadelphia, USA; 3 Department of Biomedical Sciences, Nihon University, Tokyo, Japan; 4 Graduate School of Information Sciences, Hiroshima City University, Hiroshima, Japan; 5 Laboratory for Computational Molecular Design, RIKEN Quantitative Biology Center, Osaka, Japan; 6 Hudson Institute of Medical Research, Melbourne, Australia; 7 Department of Evolutionary Study of Biosystems, SOKENDAI (The Graduate University for Advanced Studies), Hayama, Japan

**Keywords:** Sex determination, *SRY*, Molecular evolution, Marsupial mammals, Eutherian (placental) mammals

## Abstract

**Background:**

Maleness in mammals is genetically determined by the Y chromosome. On the Y chromosome *SRY* is known as the mammalian male-determining gene. Both placental mammals (Eutheria) and marsupial mammals (Metatheria) have *SRY* genes*.* However, only eutherian *SRY* genes have been empirically examined by functional analyses, and the involvement of marsupial *SRY* in male gonad development remains speculative.

**Results:**

In order to demonstrate that the marsupial *SRY* gene is similar to the eutherian *SRY* gene in function, we first examined the sequence differences between marsupial and eutherian *SRY* genes. Then, using a parsimony method, we identify 7 marsupial-specific ancestral substitutions, 13 eutherian-specific ancestral substitutions, and 4 substitutions that occurred at the stem lineage of therian *SRY* genes. A literature search and molecular dynamics computational simulations support that the lineage-specific ancestral substitutions might be involved with the functional differentiation between marsupial and eutherian *SRY* genes. To address the function of the marsupial *SRY* gene in male determination, we performed luciferase assays on the testis enhancer of Sox9 core (TESCO) using the marsupial *SRY*. The functional assay shows that marsupial *SRY* gene can weakly up-regulate the luciferase expression via TESCO.

**Conclusions:**

Despite the sequence differences between the marsupial and eutherian *SRY* genes, our functional assay indicates that the marsupial *SRY* gene regulates *SOX9* as a transcription factor in a similar way to the eutherian *SRY* gene. Our results suggest that *SRY* genes obtained the function of male determination in the common ancestor of Theria (placental mammals and marsupials). This suggests that the marsupial *SRY* gene has a function in male determination, but additional experiments are needed to be conclusive.

**Electronic supplementary material:**

The online version of this article (10.1186/s12862-018-1119-z) contains supplementary material, which is available to authorized users.

## Background

Sex determination systems are different among organisms, and flexibly evolved. In species in which male development is genetically determined, a sex-determining gene on the sex chromosomes is a dominant inducer of sex determination. The sex-determining gene is expressed in male bi-potential gonads, and enhances testis formation by regulating specific downstream genes and signaling pathways. It is believed that the origin of male-determining genes is different in mammals and fishes, and that it has independently evolved in several lineages [1–4].

In eutherian (placental) mammals such as humans and mice, the sex-determining region on Y (*SRY*) is the male-determining gene [1, 2]. The *SRY* gene is a transcription factor, and has a high-mobility group (HMG) domain (~78 amino acids). The HMG domain binds in the minor groove of specific DNA sequences, resulting in substantial DNA bending [5–7]. The protein complex of SRY and steroidogenic factor 1 (SF-1 or also called NR5A1/Ad4BP) directly binds the testis enhancer of sry-box 9 (*SOX9*) core (TESCO), and up-regulates the expression of *SOX9* [8, 9]*.* SOX9 drives testis development by mediating certain pathways, and maintains Sertoli cell specification by activating fibroblast growth factor (FGF) signaling and Prostaglandin D2 (Pgd2) signaling [10, 11]. Sertoli cells produce anti-Müllerian hormone (AMH), causing regression of the female Müllerian ducts, and facilitate spermatogenesis and the differentiation of androgen-producing Leydig cells [10].

In mammals, *SRY* has been identified in only marsupial and eutherian mammals [12, 13]. This means that *SRY* evolved in an ancestor of Theria (marsupial and eutherian mammals). This is consistent with the observation that *SRY* does not exist in Monotremata (monotremes) such as platypuses and echidnas [12, 13]. However, the function of marsupial *SRY* is not fully understood, because to date there have been no functional assays or transgenic analyses performed using the marsupial *SRY* gene. It is not clear whether the marsupial *SRY* gene has a function in male determination, nor when *SRY* obtained the male determining function during the therian evolution. In general, *SRY* is expressed earlier than *SOX9* in the male wallaby newborn as well as the human fetus [14]. Although the eutherian *SRY* gene is expressed mainly in the testis and brain, the wallaby *SRY* gene is expressed in a broad range of tissues including testis, brain, kidney and mesonephros [14–22]. It is of interest to ask whether the marsupial *SRY* gene has a function in male determination or not [23].

In this study, we clarify the sequence difference between marsupial and eutherian *SRY* genes using the currently available *SRY* sequences, and indicate how the marsupial *SRY* gene is similar to the eutherian *SRY* gene. We then identify ancestral substitutions of the *SRY* gene at the marsupial, eutherian or therian common ancestor. We test whether the marsupial *SRY* gene is able to control the expression of *SOX9* via the TESCO system in a similar way to the eutherian *SRY* gene. We also perform computational analyses of the molecular evolution and molecular dynamics, and show the evolutionary process of the therian *SRY* gene.

## Results

### The molecular evolution of the therian *SRY* gene

**Fig. 1:**
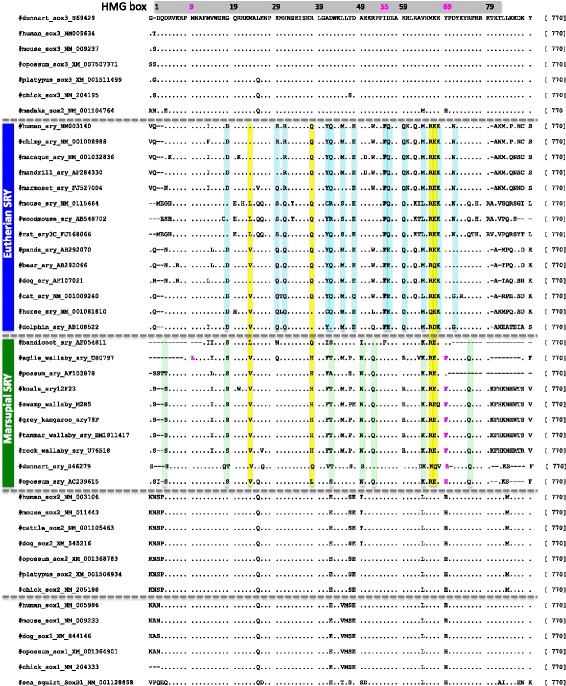
Comparison of HMG domain in *SRY* and *SOX* genes. The amino acid sequence alignment of HMG domain is shown. The gray box represents the position of the HMG domain. Eutherian-specific substitutions are indicated in blue, and marsupial-specific substitutions are indicated in green. The substitutions common to Theria are indicated in yellow. This information is also shown in Additional file 1: Table S1

**Fig. 2:**
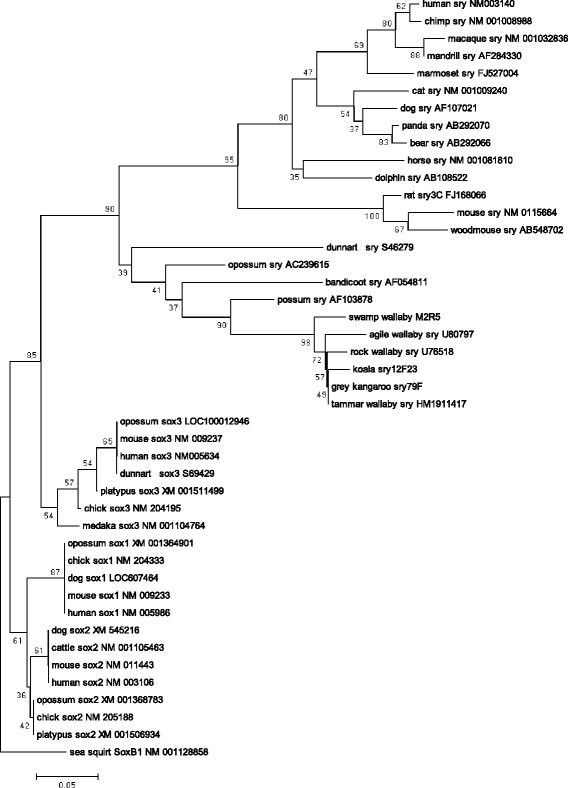
Phylogeny of *SRY* genes. The tree was constructed using the NJ method and complete deletion model (58 amino acids)

Fig. 1 shows the alignment of amino acid sequences of HMG domain in *SRY* genes. We use *SOX1, SOX2* and *SOX3* genes that are included in SOXB1 group, and it is believed that *SRY* and *SOX3* genes originated from a common ancestral gene [13, 24]. The sea squirt genome has only one gene of the SOXB1 group, and the gene is an ancestral one of the vertebrate SOXB1 group. Although the regions outside of the HMG domain are not at all aligned between marsupial and eutherian *SRY* genes, the HMG domain is highly conserved in Theria (Fig. 1). The HMG domain has ~72.8% similarity between marsupials and eutherians, and ~85.1% and ~78.7% similarity within marsupials and eutherians, respectively. We computed dn/ds using the pairwise comparison of the alignment of the entire gene, and the ratio revealed that the SRY sequence was under purifying selection (overall dn/ds = 0.16). Fig. 2 shows a NJ tree of *SRY* and *SOX1–3* genes. The topology of the tree obtained from NJ method is the same as that obtained from the other three methods (ME, MP and ML). We found that therian *SRY* genes were monophyletic, and the *SOX3* and *SRY* genes formed one cluster (Fig. 2). This means that *SRY* and *SOX3* originated from the common ancestral gene before the divergence of Theria, and it is consistent with previous studies [13, 24]. The interior branch of the stream lineage of the therian *SRY* genes is longer than that of the *SOX3* genes, although the difference of the branch length between the stem lineage of *SOX3* and *SRY* genes is not statistically significant (*P* > 0.001, Fisher’s exact test).

**Fig. 3:**
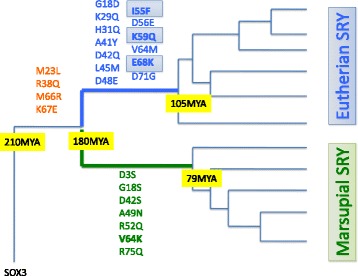
Inferred ancestral substitutions are shown on the tree by parsimony. Four substitutions were specific to the branch that *SRY* differentiated from *SOX3* in the ancestor of Theria. 13 substitutions were on the branch containing the eutherian *SRY,* and seven substitutions were on the branch containing the marsupial *SRY*. The divergence time on the tree, 79 or 105 MYA coincides with the radiation of marsupials or eutherians, and 210–180 MYA is the divergence time of monotremes and Theria

In the HMG domain*,* SRY has 48 amino acid differences compared to SOX3 (Fig. 1 and Additional file 1: Table S1). Of the substitutions, using the parsimonious method, the number of ancestral substitutions is estimated. Fig. 3 shows the ancestral substitutions that occurred in the common ancestor of Theria, marsupial or eutherian mammals, separately. Our results show that four substitutions occurred in the stem branch of Theria, and seven and 13 substitutions occurred in the stem branch of marsupial and eutherian mammals, respectively (Fig. 3 and Additional file 1: Table S1). Of the 13 eutherian substitutions, three amino acid residues (I55F, K59Q and E68K) have been conserved in the eutherian species used in this study. In marsupials, one amino acid residue (V64 K) has been conserved in the species used.

### The protein structure of the therian *SRY* gene

**Fig. 4:**
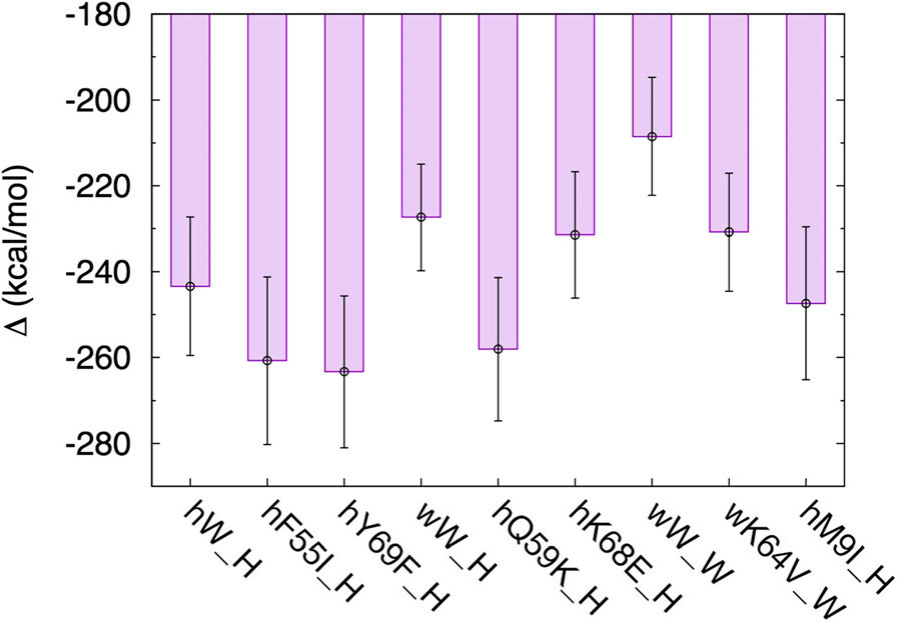
Estimated binding energy of SRY protein and DNA. The vertical axis shows the estimated binding energy (kcal/mol) of SRY proteins and DNA. The horizontal axis shows the pair of proteins and DNA. Each box length represents a mean, and each error bar represents standard deviation for 100 snapshots. The smaller values mean high affinity of proteins-DNA binding. hW_H is a pair of human wild type protein and human DNA, wW_H is a pair of wallaby wild type protein and human DNA, and wW_W is a pair of wallaby wild type protein and wallaby DNA. hF55I_H, hY69F_H, hQ59K_H, hK68E_H, and hM9I_H are pairs of human mutant type protein and human DNA. It was reported that hM9I_H shows abnormal structure (Murphy et al. 2001). wK64V_W is a pair of wallaby mutant type protein and wallaby DNA. See Additional file 1: Table S2 for more details of the pairs. The values are shown in Additional file 1: Table S4

To compare the protein structure of *SRY* genes between marsupials and eutherians, DNA binding affinities of several SRY HMG domains were investigated using the MM/PB-SA method (See methods and Additional file 1: Table S2). In the simulation we predict marsupial (wallaby) SRY-DNA structure based on the human SRY-DNA 3-D structure, and find that wallaby SRY interacts with DNA through 9 amino acid residues (Additional file 1: Table S3). The marsupial (wallaby) HMG domain can bind DNA (binding energy: −208.53 kcal/mol), but the binding affinity is a little weaker than that of the human SRY HMG (binding energy: −243.42 kcal/mol; Fig. 4 and Additional file 1: Table S4). However, it is difficult to conclude whether the wallaby SRY is functionally similar to the human SRY from these computations.

Amino acids conserved in the marsupial or eutherian mammals might affect the binding affinity, and we introduced mutations into wallaby or human amino acids in silico at positions conserved (See Material and Methods). Of the human mutant proteins, only K68E is predicted to exhibit lower binding affinity than the wild-type proteins (Fig. 4, Additional file 1: Table S4). K at the position 68 is well conserved in the eutherian SRY, although the function of the amino acid residue is unknown. The other mutant proteins in the human and wallaby rather show relatively higher binding affinity than the wild-type proteins (Fig. 4; Additional file 1: Table S4). This result suggests the conserved amino acids affect the binding affinity of SRY, but does not show that all the conserved amino acids work for higher binding affinity.

Additional file 1 Table S1 shows functionally important amino acid residues in the *SRY* gene [7, 25]. The important amino acid residues are evolutionarily conserved except for the positions 55 and 69. In human SRY, the phenylalanine (F55) and tyrosine (Y69) maintain the protein structure by anchoring the C-terminal tail and DNA-helix to the N-terminal tail, and F55 is involved in packing interactions between the N-terminal and a DNA-helix [7]. However, in the *SOX* and marsupial *SRY* genes (with the exception of the bandicoot *SRY* gene), position 55 is an isoleucine (I), (Fig. 1). The I is likely an ancestral amino acid residue, changed to F in the stem lineage of eutherian mammals (Fig. 1 and Additional file 1: Table S1). The I55F substitution is not accompanied by the change of amino acid polarity. On the other hand, at position 69, three different kinds of amino acid substitutions are observed in marsupials: F in *Diprotodontia*, serine (S) in the dunnart and histidine (H) in the opossum (Fig. 1). In this case, an ancestral amino acid residue in marsupials at position 69 is Y. Two marsupial groups of the possum and bandicoot have the ancestral residue at the position 69 (Fig. 1), thus the substitution independently occurred in the three marsupial groups. Although the function of the amino acid residue is unknown, the physico-chemical characteristic of the amino acid residue (Y) in the eutherian *SRY* is slightly different from that (F, S, or H) in the marsupial *SRY*

### The functional assay of the marsupial *SRY* gene

**Fig. 5:**
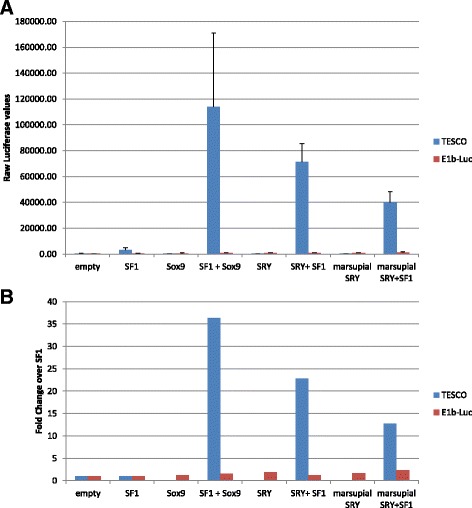
Luciferase reporter assays using SRY, Sox9, and SF1. The blue bar shows the luciferase values using TESCO-E1b-luc promoters. The red bar shows the luciferase values using only E1b-luc as a negative control. Horizontal axis indicates pairs of co-transfected vectors. (**a**) The vertical axis indicates raw luciferase values. (**b**) The vertical axis indicates fold changes over the raw luciferase value of SF1

Although we observed different patterns of substitutions in marsupial *SRY* genes from eutherian *SRY* genes and that the function (DNA-binding ability) of wallaby (marsupial) SRY and human (eutherian) SRY appears to be similar, the function of marsupial SRY is still unclear. We attempted to test whether or not marsupial *SRY* genes have a similar function to eutherian *SRY* genes as a transcriptional factor, and performed a biochemical assay of wallaby *SRY* genes in a human cell culture system of NT2/D1 cells. We investigated whether the wallaby SRY protein can activate *SOX9* transcription in the same manner as human SRY using *SOX9* enhancer reporter gene luciferase assays. In the assay, human SRY (or SOX9) with SF1 can up-regulate the luciferase expression via the mouse TESCO [9]. When we co-transfect human SRY and SF1 expression vectors into NT2/D1 cells, the luciferase activity is increased (Fig. 5). Wallaby SRY also activated *SOX9* enhancer activity, although the level of the enhancer activation by wallaby SRY/SF1 is less than that by human SRY/SF1. This suggests that function of wallaby SRY could be comparable with human SRY.

## Discussion

### The function of marsupial *SRY* genes

Whether or not *SRY* is a sex determination gene in marsupials remains debatable [12, 22, 23, 26, 27]. Our results suggest that marsupial *SRY* is functionally similar to eutherian *SRY*, and indicate that marsupial *SRY* is a male determining gene. This is the first report showing that the marsupial SRY activates the *SOX9* gene.

SRY mutations causing disorders of sex development (DSD), including gonadal dysgenesis and hermaphroditism, have been reported at 25 positions within HMG domain [25]. At those sites, most of the amino acid residues are strongly conserved, but one exception is observed at position 9 in the agile wallaby *SRY* gene (Fig. 1 and Additional file 1: Table S1). In the agile wallaby, a substitution from methionine (M) to leucine (L) has happened. An amino acid change from M to isoleucine (M9I) causes 46XY sex reversal in humans [7]. This substitution increases the angle of the bend in the DNA, and the abnormal bend may prevent distally placed proteins from interacting with a transcriptional initiation complex [7]. L and I are two of the four isomeric amino acids and the only difference between them is a position of CH_3_ in the side chain, and thus they have a similar physicochemical characteristic. We expect the substitution of I instead of L have a similar effect. This suggests that the SRY protein might not be functional during male determination in the agile wallaby.

### The perspective for molecular coevolution of the *SRY* gene

The evolution of *SRY* probably consisted of two steps. The first step is the differentiation from *SOX3* to *SRY* in the common ancestor of Theria. Our study estimates four substitutions in the stem lineage of Theria (during ~30 million years). Indeed, the tempo of the amino acid substitutions in the therian ancestor is significantly faster than that in the eutherian or marsupial ancestor (Fisher’s exact test; *P* < 0.001). This suggests a possibility of the functional diversification of *SRY* from *SOX3* in the therian ancestor, but the biological meaning of the four substitutions or functional diversification is unknown. A previous study showed that ectopic *SOX3* could activate *SOX9* in the same way as *SRY* in mice [28]. Therefore, *SOX3* might have a potential function for male determination, but as *SOX3* is not expressed in the developing testis this precludes it from working as a male determination gene in nature. The changes of the expression pattern were necessary [29], so that the ancestral *SOX3* could reach the top of the sex determination system as *SRY*. Moreover, the differentiation of protein sequences was essential for the therian ancestral gene to acquire the *SRY* functions*.*

The second step in the evolution of the *SRY* gene caused the functional difference between the marsupial and eutherian genes. In the eutherian ancestor, SRY probably obtained the ability to functionally interact with SF-1 and *SOX9,* and the SRY and SF-1 proteins could bind the *SOX9* enhancer (TESCO)*.* Our result indicates that the marsupial *SRY* can use the eutherian TESCO, but the TESCO sequence does not exist in marsupials [8, 30]. The upstream region of *SOX9* in marsupials has only partial consensus sequences of the SRY and SF-1 binding sites, although there is a possibility that marsupials have a testis enhancer of *SOX9* at a different position. We also found a difference in amino acid sequences between the marsupial and eutherian *SRY* genes*,* and the marsupial *SRY* interacted with DNA weakly compared to eutherian *SRY.* The sequence differences between the marsupial and eutherian *SRY* might affect the stability of protein-DNA complex.

The evolution of *SRY* is associated with the sub-functionalization of duplicate genes. One of the duplicate genes accumulates mutations, and sometimes reinforces one of the functions that the ancestral gene has [31]. Although *SOX3* and *SRY* are homologous non-recombining genes shared between the X and Y called gametologs, *SOX3* is the ancestral gene, and *SRY* is the reinforced new one. The sub-functionalization of *SRY* might be involved in leading to the emergence of sex chromosomes in Theria.

## Conclusion

In this study, we showed that the marsupial *SRY* could work as a transcription factor in a human cell culture system, and could substitute for human *SRY* in the TESCO assay. Our study suggests that the marsupial *SRY* is functionally similar to the eutherian *SRY*. We found several sequence differences between the marsupial and eutherian *SRY* genes, and these sequence differences support functional differences between the marsupial and eutherian *SRY* genes including a different range of DNA-binding affinity in the proteins.

## Materials and methods

### Nucleotide sequences and animal samples used in the analysis

Nucleotide sequence data and corresponding gene information were obtained from NCBI (http://www.ncbi.nlm.nih.gov) and Ensembl databases (release 62; http://uswest.ensembl.org/index.html). The datasets used and/or analyzed during the current study are available from the corresponding author on reasonable request. A BLAST search was carried out using the human or wallaby *SRY* genes as a query to identify *SRY* homologs in eutherian and marsupial species. We found *SRY* sequences from 72 species (49 eutherians; 23 marsupials), but after exclusion of the same sequences in closely related species, 14 sequences from eutherians and ten sequences from marsupials were compared for coding region sequences. Of the ten marsupial SRYs, seven were published: brush-tailed possums (*Trichosurus vulpecula*), northern brown bandicoot (*Isoodon macrourus*), agile wallaby (*Macropus agilis*), tammar wallaby (*Macropus eugenii*), brush-tailed rock wallaby (*Petrogale penicillata*), striped-faced dunnart (*Sminthopsis macroura*) and opossum (*Monodelphis domestica*), and three were obtained in this study as follows.

We experimentally identified three marsupial *SRY* homologs (accession numbers: LC111530; LC111531; LC111532) because published *SRY*s are limited and some are truncated. Liver and spleen samples were collected from swamp wallabies (*Wallabia bicolor*), eastern gray kangaroos (*Macropus giganteus*), and koalas (*Phascolarctos cinereus*). Those samples were donated from Kanazawa Zoo in Yokohama City, Japan, and informed consent for use of the samples was written. Genomic DNA was isolated using the DNeasy Blood & Tissue Kit (QIAGEN). Genomic DNA (100 ng) was suspended in 50 μl of 1×Ex Taq PCR buffer, which contained 0.2 μM of each deoxyribonucleotide triphosphates (dNTP), 0.5 μM of the one pair of primers, and 1 unit of TaKaRa Ex Taq DNA polymerase (TaKaRa). The oligonucleotide primers used are F-TTGAGTCCGTGAAAAGTGGGTC and R-TTGTGAATCTGCCACGCTTGTC for swamp wallabies and koalas and F-GCTATGTATGGCTTCTTGAATG and R-AACTGTCATTCGTTTCAGGT for eastern gray kangaroos [22, 32, 33]. Polymerase chain reaction (PCR) amplification included one cycle at 95 °C for 30 s followed by 30–40 cycles of denaturing for 15 s at 95 °C, annealing for 30–60 s at 50–60 °C, and extension for 60 s at 72 °C. A final extension was performed for 10 min at 72 °C. PCR products were purified using the QIAquick Gel Extraction Kit (QIAGEN), or they were subcloned using the TOPO XL PCR cloning kit (Invitrogen). In the case of direct sequencing, PCR products were purified with ExoSAP-IT (United States Biochemical) for 30 min at 37 °C followed by 15 min at 80 °C. Those purified products were sequenced. The sequencing reactions were performed using the dideoxy chain-termination method [34] using BigDye Terminator v1.1 or 3.1 Cycle Sequencing Kits (Applied Biosystems), and the sequencing reactions were analyzed on an Applied Biosystems 3130 genetic analyzer.

### Phylogenetic and data analyses

The nucleotide sequences were aligned using Clustal X [35], and the results were also checked manually. The entire alignment is available upon request. Phylogenetic trees were constructed using all four methods available in the MEGA4.1 program [36] and MEGA6.06 [37]: neighbor-joining (NJ) [38], minimum evolution (ME) [39], maximum parsimony (MP) [40] and maximum likelihood (ML) [41]. The reliability of the trees was assessed by bootstrap re-sampling with 1000 replications. Phylogeny inference package version 3.68 (PHYLIP) [42] and phylogenetic analysis by maximum likelihood (PAML) [43] were also used to construct a phylogeny based on ML.

### Details of molecular dynamics (MD) simulations

We performed MD simulations of 9 systems to analyze the binding affinity of human- or marsupial-DNA to *SRY* proteins. The DNA-protein pairs used in this analysis are shown in Additional file 1: Table S2. 6 mutant proteins were selected in order to understand the functional importance of an amino acid residue that was evolutionary conserved in the eutherian or marsupial *SRY* genes. The mutant proteins had a point mutation at the conserved amino acid residue, and we investigated the binding affinity between the mutant proteins and DNA. At positions 55, 59 and 68, three amino acid residues are eutherian-specific and evolutionary conserved in all the eutherians used. An amino acid residue at a position 64 is marsupial-specific and conserved in all the marsupials used. These amino acid residues might be important for a function in the *SRY*, and were changed to the ancestral one in silico. At a position 69, an amino acid residue (Y) is conserved well in *SOX3* and *SRY* except for some marsupials (*Diprotodontia*, the dunnart and opossum). The amino acid residue might be important in the eutherian but not in the marsupial one. The amino acid residue (Y) in the human protein was changed to the *Diprotodontia* (wallaby) one (F). The amino acid residues at positions 55 and 69 are also essential to the human protein structure (Additional file 1: Table S1). The detail of M9I mutation was explained in Discussion [7]. The regulatory region of the human *Amh* was used as SRY protein binding sequence [7]. In addition, marsupial SRY binding sequence was used for the MD simulations and was identified from the 5′ regulatory regions of the wallaby and opossum *SRY* genes using MatInspector [44]. All initial structures were modeled by using modeling software MOE (Chemical Computing Group Inc.) based on nuclear magnetic resonance structure of human SRY HMG domain bound to a 14 nucleotide sequence (PDB ID: 1 J46) [7]*.* For the mutant proteins, the mutated residues were replaced manually by using MOE. These initial structures were solvated with explicit water molecules in a rectangular box, and counter ions (Na^+^ and Cl^−^) were added for neutralizing the protein’s charge. The minimum distance between a protein atom and the water wall was 12 Å. We did the energy minimization of the entire system, and then gradually increased the system’s temperature to 310 K using the Berendsen thermostat [45]. For each system, a 50-ns production run in the NPT ensemble was performed. The temperature of the system was controlled at 310 K using a Langevin thermostat. All simulations were carried out by using AMBER software package version 12 [46]. The simulations were done using a periodic boundary condition, and the long-range electrostatic interactions were treated using the particle-mesh Ewald method [47, 48]. We applied the ff99SB-ildn-NMR force field [49, 50] for amino acids and TIP3P model [51] for water molecules. All bonds involving hydrogen atoms were constrained by the SHAKE [52] and SETTLE [53] algorithms, and the time step of 1 fs was used.

### Analysis of binding affinity between DNA and proteins

The DNA-binding affinity of HMG domains from eutherian, marsupial, or mutant *SRY* proteins was investigated from the trajectories of MD simulations. The binding energies between proteins and DNA were estimated by using the molecular mechanics/Poisson-Boltzmann and surface area (MM/PB-SA) method [54–58]. In MM/PB-SA method the binding free energy is calculated as

1 $$ \Delta {G}_{\mathrm{binding}}=G\left(\mathrm{complex}\right)-\left\{G\left(\mathrm{apoprotein}\right)+G\left(\mathrm{freeligand}\right)\right\} $$

2 $$ G\left(\mathrm{molecule}\right)=\left\langle {E}_{MM}\right\rangle +\left\langle {G}_{\mathrm{solvation}}^{\mathrm{polar}}\right\rangle +\left\langle {G}_{\mathrm{solvation}}^{\mathrm{nonpolar}}\right\rangle - TS $$

3 $$ \left\langle {E}_{\mathrm{MM}}\right\rangle =\left\langle {E}_{\mathrm{internal}}\right\rangle +\left\langle {E}_{\mathrm{electrostatic}}\right\rangle +\left\langle {E}_{\mathrm{vdW}}\right\rangle $$

4 $$ \Delta {G}_{\mathrm{solvation}}^{\mathrm{nonpolar}}=\gamma A+b $$

In the analysis of the binding energies, the water molecules were replaced with implicit solvation models. The conformations of the apoprotein (SRY) and free ligand (DNA) were extracted from the set of structures of protein/DNA complex for calculating *G*(apoprotein) and *G*(free ligand) in Eq. (1). < > denotes an average over a set of structures along an MD trajectory. *E*_internal_ includes the bond, angle, and torsion angle energies, and *E*_electrostatic_ and *E*_vdW_ are intermolecular electrostatic and vdW energies, respectively. The *G*_solvation_^polar^ was calculated by solving the Poisson-Boltzmann equation with Delphi program [59]. The dielectric constants for the solute and surrounding solvent were 1.0 and 80.0, respectively. In this study the entropy was not calculated. The MD trajectory was collected for a 30 ns (from 20 to 50 ns) with a time step of 300 ps for each system.

### Cell culture and transfections

We evaluated the function of marsupial SRY in a human embryonic carcinoma cell line, NT2/D1, as a model of presumptive Sertoli cells [9]. Ethics approval is not necessary for research use of cell lines within Australia. NT2/D1 cell-lines (obtained from ATCC CRL-1973) were grown as an adherent monolayer in DMEM:F12 (GIBCO) supplemented with 10% fetal calf serum (FCS) (GIBCO) and 5% penicillin/streptomycin and were incubated at 37 °C in 5% CO_2_ in a humidifying incubator (NU AIRE). Cells were seeded in 12-well culture plates at a density of 200,000 cells per well. Cells were grown for 24 h before the transfection of DNA. Transient transfections were conducted using *X-tremeGENE 9* DNA Transfection Reagent (Roche). The protocol required the use of a ratio of *X-tremeGENE 9* DNA Transfection Reagent to DNA of 3:1. Plasmid DNA was added to *X-tremeGENE 9* in growth media and incubated for 20 min before addition to cells. Cell lysates were harvested after 48 h for reporter assays.

### Expression and reporter plasmids

Expression vector is a plasmid of pcDNA3 origin (Clontech) for all mammalian genes. Marsupial SRY ORF was amplified by PCR from tammar wallaby male genome DNA from Water Paul in Australian National University using a forward primer, ATCATAGATCTGCCACCATGTACCCATACGATGTTCCGGATTACGCTAGCCATATGTATGGCTTCTTGAATGTA, which is containing BamHI, FLAG and KOZAK sequences and a reverse primer, TTAGAAACTGTCATTCGTTTC, which replaces the stop codon with an EcoRI restriction site [9, 60]. The marsupial SRY clone was sub-cloned into pcDNA3. The mouse TESCO sequence was sub-cloned into E1b-luciferese vector [9]. Each HA-tagged human SF1 and SOX9 expression plasmid is previously described [9, 61]. It notes that a marsupial-originated plasmid is only SRY clone, but other SF1/SOX9/TESCO plasmids originate from human or mouse sequences because the sequences are highly conserved in Theria [8, 30, 62, 63].

### Luciferase reporter assays

At 48 h post-transfection the culture media was removed from 12-well plates and cells were washed twice with 100 μl PBS per well. 100 μl of 1 x Reporter Lysis buffer (Promega) was added to each well and placed onto a shaker at room temperature for 10 min. Plates were then tapped gently to lift cells. Lysed cells were then collected and placed into 1.5 ml Eppendorf tubes. Cell lysates were centrifuged for 10 min at top speed, and supernatant was used in reporter assays. For Luciferase reporter assays, 100 μl of the cell extract was added to a 96-well Luciferase plate. 100 μl of 2X Luciferase assay buffer was then added to each well. Luminescence readings at 405 nm were instantly measured, and Luciferase activity was determined. Statistical significance was determined using two-tailed unpaired Student’s T-test.

## Additional file

Additional file 1: Table S1. The functionally important amino acid residues and substitutions in SRY. **Table S2.** The pair of proteins and DNA used for the molecular dynamics analysis. **Table S3.** The list of amino acid residues interacting with DNA. **Table S4.** The values of the binding energy of protein and DNA. (XLSX 17 kb)

## Abbreviations

3-D: three-dimensional
AMH: Anti-Müllerian hormone
DSD: Disorders of sex development
F: Phenylalanine
FGF: Fibroblast growth factor
H: Histidine
HMG: High-mobility group
I: Isoleucine
MD: Molecular dynamics
ME: Minimum evolution
ML: Maximum likelihood
MM/PB-SA: Molecular mechanics/Poisson-Boltzmann and surface area
MP: Maximum parsimony
NJ: Neighbor-joining
PAML: Phylogenetic analysis by maximum likelihood
Pgd2: Prostaglandin D2
PHYLIP: Phylogeny inference package
S: Serine
SF1: Steroidogenic factor 1
SOX: Sry-box
SRY: Sex-determining region on Y
TESCO: Testis enhancer of Sox9 core
Y: Tyrosine
